# The Impact of Hyperarch Fascia Training on Perceived Ankle Instability and Vertical Jump Performance in Physically Active Men and Women

**DOI:** 10.3390/sports14100468

**Published:** 2026-10-20

**Authors:** Kenneth B. Hawkins, Chong Xie, Cristal Abreu Matias, Eric M. Sikorski, Gabriel J. Wilson, Charlie Ottinger, Ryan Lowery, Jacob M. Wilson

**Affiliations:** 1 Applied Science and Performance Institute, Tampa, FL 33634, USAcabreu@theaspi.com (C.A.M.); esikorski@theaspi.com (E.M.S.); gwilson@theaspi.com (G.J.W.); cottinger@theaspi.com (C.O.); rlowery@theaspi.com (R.L.); 2 Hyperarch Fascia Training, Roslyn, NY 11576, USA; info@secret-of-athleticism.com; 3 School of Health Sciences, Barton College, Wilson, NC 27893, USA

**Keywords:** chronic ankle instability, fascia, myofascia, ankle function, performance, vertical jump, countermovement jump, barefoot, barefoot training, meditation, mental imagery

## Abstract

Chronic ankle instability (CAI) is a prevalent musculoskeletal condition that impairs physical performance and increases reinjury risk; yet, training programs emphasizing fascia-targeted techniques remain an understudied rehabilitation strategy. The purpose of this study was to examine the effects of 12 weeks of a multicomponent training intervention emphasizing fascia-targeted techniques (Hyperarch Fascia Training (HFT)) on perceived ankle instability and function, and vertical jump performance in individuals with CAI. In this decentralized, randomized controlled trial, 66 participants were randomized (HFT *n* = 44, CON *n* = 22), with 43 participants completing the study and being analyzed (HFT *n* = 22; CON *n* = 21). Selected subjects confirmed perceived ankle instability (CAIT score ≤ 25) and were assigned to either a structured, multicomponent HFT program with various foot and ankle strengthening exercises, or a passive control condition instructed to maintain activities of daily living. The HFT group experienced disproportionately higher attrition (*n* = 22 excluded from analysis) than CON (*n* = 1 excluded from analysis due to becoming unresponsive). The reasons for attrition in the HFT group were due to becoming unresponsive (*n* = 13), self-reported difficulty with the protocol (*n* = 3), scheduling conflicts (*n* = 3), or adverse events (*n* = 3, reported as moderate). Outcomes were assessed at baseline, week 8, and week 12 using the Cumberland Ankle Instability Tool (CAIT) (primary outcome), Foot and Ankle Ability Measure (FAAM) (primary outcome), countermovement jump (CMJ) (secondary outcome), and Short Form Health Survey 12 (SF-12) (exploratory outcome). The HFT group demonstrated significantly greater improvements in CAIT scores compared to CON (group × time interaction *p* = 0.008), with large within-group effect sizes in the HFT group relative to baseline (d = 1.49 and 1.54, at weeks 8 and 12, respectively). Significant group × time interactions also favored HFT for CMJ jump height (*p* = 0.017) and flight time (*p* = 0.005) (co-secondary outcomes), as well as the CMJ algorithmic derivatives of mean velocity (*p* = 0.001) and take-off velocity (*p* = 0.008) (exploratory outcomes). No significant group × time interactions were observed for FAAM ADL or Sport subscales. Significant group × time interactions were found for SF-12 survey results for Physical Function (*p* = 0.045), Limits due to Physical Health (*p* = 0.030) and General Health (*p* = 0.043), with CON tending to decline over time and HFT tending to improve. The HFT group was higher than CON on Physical Function at Week 8 (d = 1.04, *p* = 0.009), and on Perceived Pain at Week 12 (d = 0.88, *p* = 0.047). Overall, these findings suggest that a multicomponent fascia-centered training program may represent a viable intervention for improving perceived ankle stability and increasing the explosive vertical jump performance in individuals with CAI.

## 1. Introduction

Chronic ankle instability (CAI) is the principal long-term consequence of lateral ankle sprain and is among the most frequently sustained musculoskeletal injuries in physically active populations [1]. Acute sprains reoccur at high rates, and recurrence is strongly associated with progression to CAI and to post-traumatic osteoarthritis [2]. Supervised rehabilitation is the accepted first-line management. Consistent with this, a network meta-analysis of 21 randomized trials reported that multimodal programs combining balance training, strengthening, functional tasks, and range-of-motion work improved self-reported instability of the Cumberland Ankle Instability Tool (CAIT) relative to the control [3,4]. Eligibility criteria and participant characteristics remain inconsistently reported across this literature, limiting comparison between trials [5].

A specific component of strength and conditioning programs is injury rehabilitation, which often entails interdisciplinary practice with athletic trainers and/or physical therapists. Proper injury rehabilitation is a primary outcome of this collaborative practice, as injured tissue is more likely to be reinjured [6] and unresolved injuries can result in upstream biomechanical compensations that impair performance or expose other joints/tissues to greater injury risk [7]. A number of exercise training variables have been promoted as potential injury rehabilitation options, such as isometric contractions [8], loaded eccentric muscle actions [9], and even strategies such as blood flow restriction training [10]. Each methodology adheres to the principle of specificity in that specific stimuli are applied to the musculoskeletal tissue to generate distinct recovery pathways. Quick and effective injury rehabilitation is of great interest to physically active individuals, practitioners, and all involved stakeholders; thus, research is continually assessing novel approaches for achieving optimal outcomes.

A unique and relatively novel exercise regimen involves targeted training of the myofascial tissue. Myofascia, or simply “fascia,” is often described as a connective tissue framework that surrounds skeletal muscle, connective tissue, bones, and even organs, thus creating a continuous web of interconnected tissue throughout the body [11]. As a unique exercise system, fascia training focuses on distinct contraction and stretch patterns to stimulate muscle and fascial tissue [11,12] alongside fascial manipulation through interventions such as manual therapy, foam rolling, etc. [13]. These variables may be applied through different degrees of stretching and stretch velocities or specific fascial disruption techniques that are intended to activate the numerous mechanoreceptors within the fascia’s collagen network [14]. Activation of these receptors provides a fascia-specific mechanotransduction pathway, which may result in architectural adaptive changes to the fascial tissue [15]. Importantly, this adaptive pathway may not require the same threshold for musculoskeletal remodeling, meaning that fascia tissue adaptation may require a lower-intensity mechanical or biochemical stimulus [16,17], which is important in the context of injury recovery or rehabilitation. Despite these theoretical outcomes, the role of fascia-specific training and fascial adaptation is less understood in the strength and conditioning field, as the adaptive potential of the fascia system likely differs from the muscular and neuromuscular systems [12]. Moreover, the overall output of the musculoskeletal system is influenced by fascial performance [18], so gaining an understanding of fascial training outcomes is of paramount importance to strength and conditioning practitioners. Unfortunately, to date, there are few published studies that have reported on the effects of fascia training programs.

Injuries and conditions such as tendonitis, iliotibial band syndrome, shin splints, and plantar fasciitis are all considered fascial injuries [19]. Recently published studies, including Mohammadi et al. 2024 [14] and Maetzler et al. 2020 [20], examined the effects of a specific fascia training method termed the fascia disruption model (FDM) as proposed by Typaldos 2002 [21]. The FDM posits that injury pain and/or non-specific joint pain may be related to alterations in the fascia/connective tissue system. The method provides specific interventions to correct the disruptions, including manual therapies such as trigger point massage, joint mobilization/distraction, etc. The aforementioned research studies found that fascia training, specifically using the FDM, was effective at enhancing joint range of motion in patients suffering from chronic ankle pain [13] as well as improving balance in subjects with previous ankle injuries [13,20]. Studies have also reported acute improvements in dynamic balance, with Pawik et al. 2021 [22] providing a single myofascial manual therapy session to patients with quadriceps femoris injuries. Finally, Brandolini et al. 2019 [23] reported a significant decrease in ankle injuries in professional athletes following fascial manipulation, which emphasizes deep friction manipulation at specific musculoskeletal sites, thus presenting a unique approach compared to the FDM. Ultimately, research into fascia training lacks a clear consensus for effective practices, specific techniques, or evidence-based practitioner guidelines and, therefore, remains an intriguing but understudied training method for injury rehabilitation.

Therefore, the purpose of this study was to examine the effects of 12 weeks of a multicomponent training intervention emphasizing fascia-targeted techniques (Hyperarch Fascia Training (HFT)) (New York, NY, USA) on subjects with previous ankle injuries and chronic ankle instability (CAI). Outcomes measures included objective and subjective markers of perceived ankle function, health, and jump performance. We hypothesized that HFT would be more effective than a control group at enhancing perceived ankle function, vertical jump performance, and subjective confidence within sporting tasks and activities of daily living.

## 2. Materials and Methods

### 2.1. Study Design and Protocol Overview

This study was conducted as a 12-week decentralized clinical trial across the United States, using a randomized design (Supplementary Materials Table S1: CONSORT checklist) to evaluate the potential efficacy of HFT in reducing pain and improving functional performance in individuals with a history of chronic ankle sprains, in either the HFT group or the control (CON) group. Specifically, the first primary outcome measure evaluated ankle function and instability using the widely validated Cumberland Ankle Instability Tool (CAIT). The additional primary measure assessed functional ankle ability using the Foot and Ankle Ability Measure (FAAM). Additionally, as a secondary measure, participants performed a countermovement jump assessment (CMJ), which was used to capture subject jump height and flight time (co-secondary outcomes) as an objective assessment of lower limb functional mobility, with additional algorithmically estimated outcomes of force, relative force, power, relative power, mean velocity, take-off velocity, and impulse also presented as exploratory outcomes. Finally, as an exploratory measure, subjects were required to complete the short-form health survey (SF-12) for indication of general perceived health and quality of life throughout the course of the trial. The endpoint for all post-outcome assessments was the week-8 and -12 timepoints. The protocol was approved by an external Institutional Review Board (IRB) (Advarra; Columbia, MD, USA; Protocol #Pro00085874; approval date: 17 April 2025) and was prospectively registered with ClinicalTrials.gov (ID: NCT06959472) (28 April 2025) prior to the onset of participant recruitment (5 May 2025).

Before engaging in any study procedures, all of the participants were screened using the inclusion and exclusion criteria, including medical history. Screening included the self-reported duration of ankle injuries or challenges, in months or years, to confirm a minimum symptom duration of at least 6 months, as well as screening for injuries to the Achilles, plantar fascia, hip, or turf toe within the preceding 6 months, which served as an exclusion criterion. Subjects had no public involvement in the design, conduct, and reporting of the trial or in developing plans for recruitment, design, or implementation of this study. No subjects were asked to advise on the interpretation or writing up of results.

Randomization was performed after completion of baseline assessment, and stratified by CAIT score (≤25, confirming chronic ankle instability in at least one foot), sex, and age. The allocation sequence was generated using Microsoft Excel. Allocation was implemented by members of the research team who were also involved in participant enrollment, and allocation was therefore not concealed from the enrolling investigators. The trial was unblinded to participants, coaches, and investigators throughout, as blinding was not feasible with a supervised exercise intervention. After approximately the first month of recruitment, it was recognized that the HFT group experienced substantially higher attrition than CON, attributable to the difficulty of the protocol, scheduling conflicts, or loss of contact with participants. The allocation ratio was therefore changed to 2:1 in favor of HFT, in order to preserve an adequate number of HFT completers. This was an adaptive change made during recruitment rather than a prespecified design feature. Of the 66 participants randomized, 22 were allocated to CON and 44 to HFT. Twenty-three participants did not contribute outcome data after randomization (CON *n* = 1, 4.5%; HFT *n* = 22, 50.0%), giving an analyzed sample of 43 (CON *n* = 21; HFT *n* = 22). The analysis population comprised all randomized participants contributing at least one observation after randomization, utilizing a modified intention-to-treat analysis. Baseline comparability between groups is described using standardized mean differences (Table 1a,b). Baseline characteristics of completers and non-completers within the HFT arm differed only modestly (age d = 0.25, BMI d = 0.36, sex d = 0.10, height d = 0.07). Standardized mean differences between groups were most notable for BMI (0.79), age (0.58), and weight (0.55), and were no greater among the 43 analyzed participants than among the 66 randomized.

Following successful screening, continued eligibility, and completion of the baseline assessment, participants were instructed to commence their assigned intervention. The primary outcome metric of CAIT collected from both groups at the baseline, 8 weeks, and 12 weeks pertained to the indexed ankle, which was identified by their CAIT score collected at baseline testing (described in Section 2.3). Participants in the CON group were told to maintain their normal activities of daily living for the full 12-week period. They were instructed to refrain from initiating any new exercise programs, significant dietary changes, travel, or altering lifestyle behaviors that could influence study outcomes. The HFT group participated in a proprietary, structured multicomponent 12-week foot and ankle training program led by an assigned HFT coach targeting fascial health through combined physical and mental training. In each session, participants engaged in a one-hour virtual training session led by a certified hyperarch fascia training (HFT) coach, who instructed the participants on proper form and execution (Supplementary Materials Table S4: Hyperarch fascia training protocol). Workouts consisted of approximately 2–3 sets each until volitional fatigue, or approximately 1–2 min of work, of myofascial rolling, elevated towel curls, toe raises, single-leg balance exercises, plyometric activities, mental imagery, and movement drills, all performed barefoot. Regarding the specifics of these exercises, for myofascial rolling, HFT coaches used the meridian lines as the main targets, as they integrate with body functions, starting from the foot, proceeding to the calf and glute, and progressing along the kinetic chain. For balance exercises, coaches started with standing on two feet, until the proper posture was achieved, then progressed to a single-leg balance with one foot while the other foot was holding a marble and moving in a different plane of motion. Likewise, plyometric jumping activities started with both feet and progressed to single-leg activity, with the instructions on form given through jumping movements. Regarding mental imagery, subjects were instructed to imagine their body as one unit and move like one, with a specific focus on moving the energy from the feet upwards to the body. Lastly, movement drills consisted of moving two feet in all directions, with a particular emphasis placed by coaches on moving from heel to toes. Then, once subjects were deemed by the coaches to have achieved good balance, control and rhythm in transitioning to the balls of their feet, subjects progressed to landing using only the balls of their feet.

The features that distinguish the present program are its exclusively barefoot delivery, its emphasis on the foot–ankle interface as the organizing principle of the session, its integration of mental imagery, and its deliberate exclusion of loaded triple-extension exercises. HFT subjects were re-enforced via weekly live virtual sessions with the coaches, as well as two additional approximately one-hour independent practice sessions per week, totaling approximately 3 h per week of overall participation for individuals assigned to that study group. The independent sessions consisted of practicing and replicating the exercises they performed during the guided coaching sessions.

Regarding compliance for study subject completers, HFT coaches confirmed full compliance with the protocol, with the subjects attending the virtual sessions and completing their individual assigned work as well.

**Figure 1:**
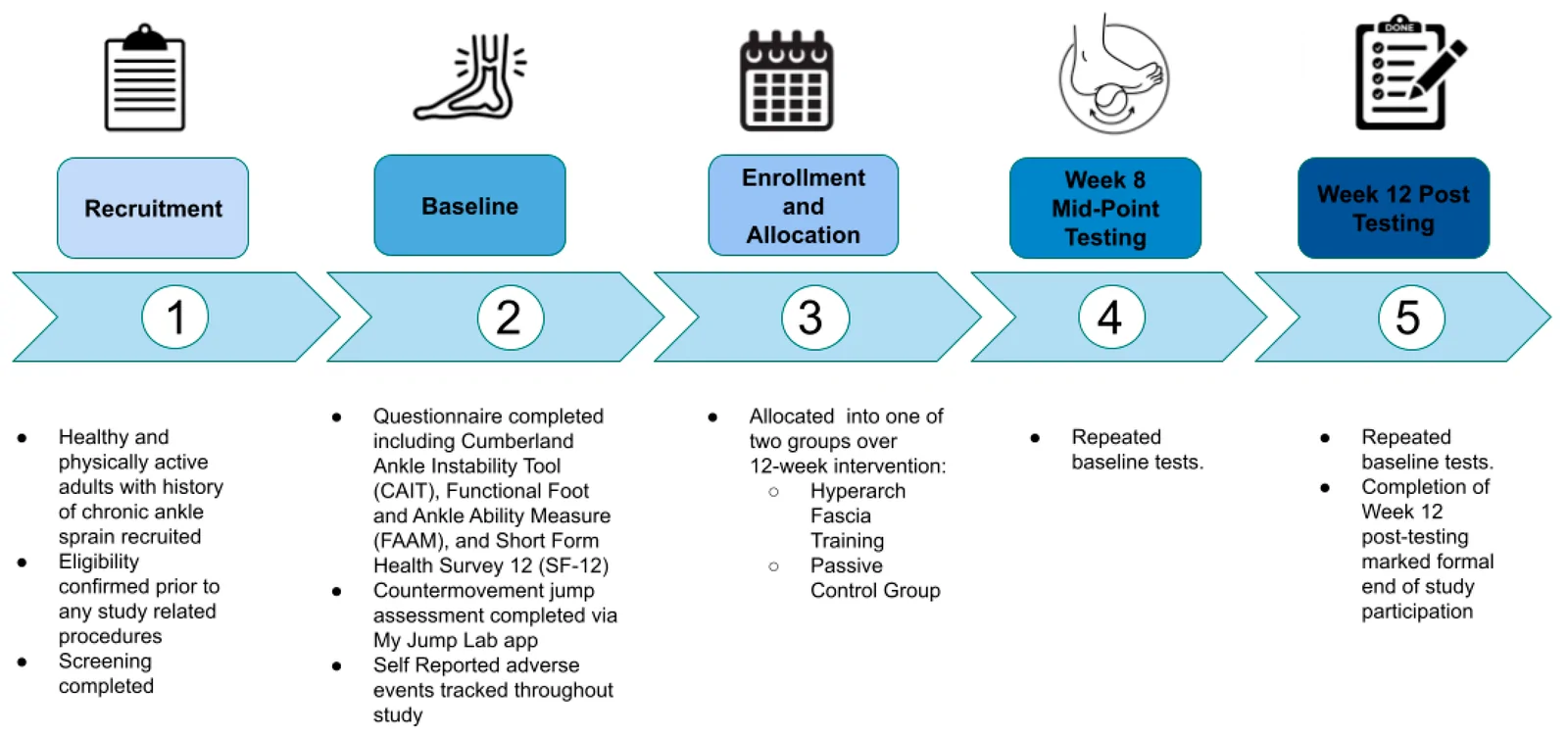
Study design infographic. The arrows and numbers are listed chronologically in order of study execution, from the left to the right.

Study design is shown in Figure 1. Adverse events were monitored through communication via email as well as upon questionnaire completion at baseline, at post-8-week assessments, and at post-12-week assessments. Completion of all post-testing assessments marked the formal conclusion of study participation.

### 2.2. Study Participants

Sample size was determined using an a priori power analysis (G*Power, version 3.1), based on calculated effect sizes (f = 0.59) from the results of Pawik et al. 2020 [22]. The calculation assumed an effect size of f = 0.59, a two-sided alpha of 0.05, and statistical power of 0.95 (1 − β), for repeated-measures within/between ANOVA with two groups and three measurements. The results of the power analysis suggested approximately 10 subjects per group to detect significant differences between groups. As this was a first-time study for this particular multicomponent intervention, we utilized a more conservative sample size target goal of finishing with ~20 subjects per group (~40 total). To achieve this, we aimed to recruit ~50 subjects in total, accounting for dropouts. We note that an effect size of f = 0.59 is substantially larger than is typical for exercise interventions, and that it was derived from a different intervention in a different population; it should therefore be regarded as an optimistic assumption rather than an evidence-based projection for the present intervention, and the conservative recruitment target was adopted for this reason. Sensitivity analysis for the realized sample of 43 participants indicates that the design provides 80% power to detect a group × time interaction of f ≥ 0.17, assuming a correlation among repeated measures of 0.65, but only 36% power to detect a between-group difference of d = 0.50 at any single visit, with 80% power reached only at d ≈ 0.88. The trial is therefore adequately powered for the interaction tests on which its conclusions rest, and underpowered for between-group comparisons of small to moderate magnitude at individual timepoints.

**Figure 2:**
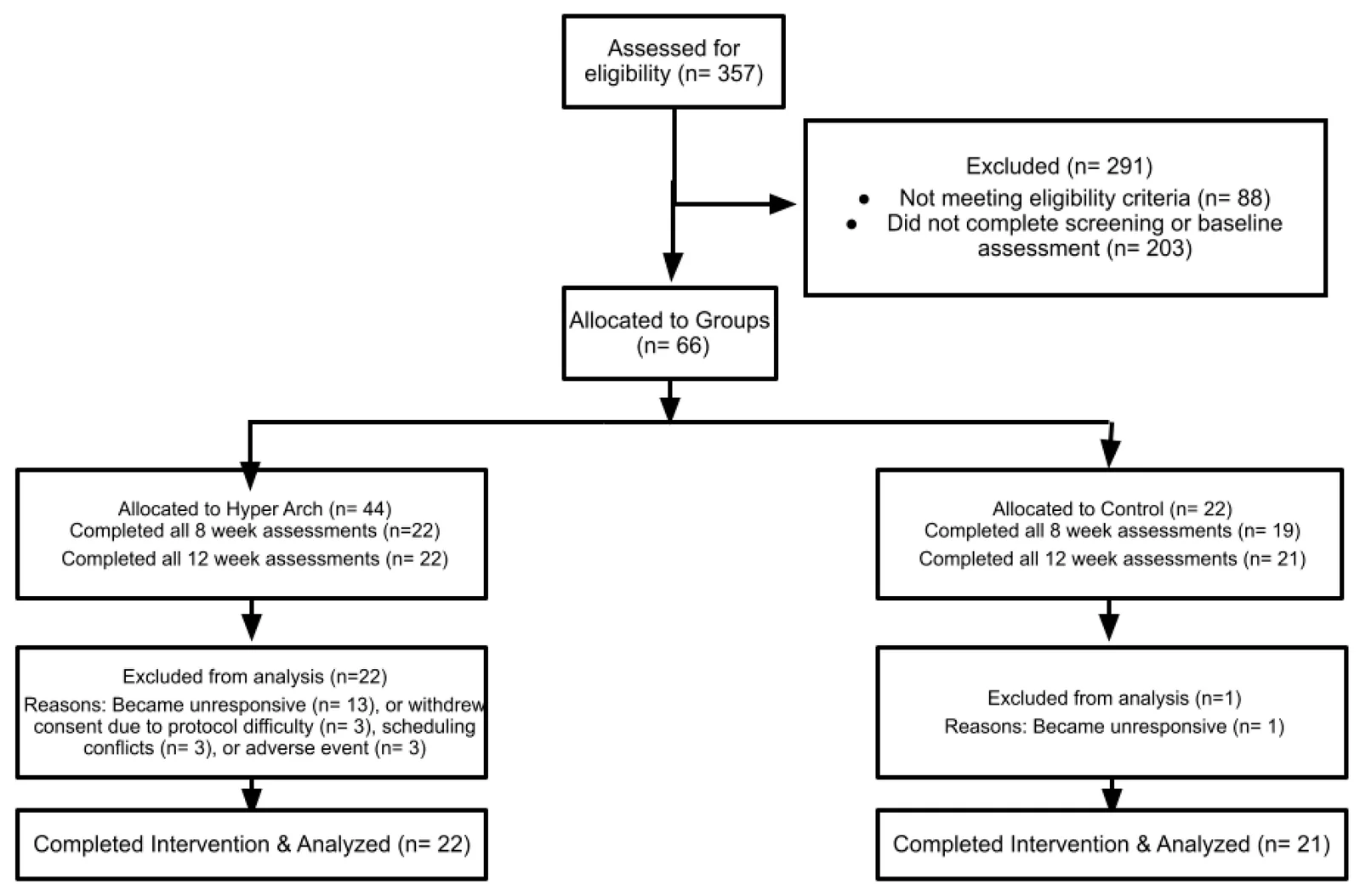
CONSORT flow diagram.

Recruitment began on 5 May 2025 with the distribution of initial eligibility screening emails, and the first participant was formally enrolled on 7 May 2025, both of which followed IRB approval (17 April 2025) and clinical trial registration (28 April 2025). Participant eligibility was confirmed through the CAIT score, medical history and inclusion/exclusion screening. Mandatory Zoom calls were scheduled to review all the informed consent, after which participants signed the informed consent form electronically and completed the baseline assessment battery. Only participants who successfully completed baseline testing were then assigned to either the HFT or CON group. A total of 357 physically active participants were screened for eligibility according to the inclusion and exclusion criteria (Supplementary Materials Table S2: Study eligibility criteria). Male and female participants, aged 20–45 years old, were recruited via the User Interviews platform (User Interviews, Inc., Brooklyn, NY, USA). A total of 66 participants signed the ICF and were randomized and allocated to a group in an ~2:1 ratio, with 44 subjects allocated to the HFT intervention group and 22 allocated to the passive CON group. Of the 44 participants randomized to HFT, 22 (50%) did not complete this study. This may be compared with 1 of 22 randomized CON participants (4.5%), who became non-responsive during their completion of study visits after the baseline. After reviewing individual withdrawal notes, HFT attrition was attributable to becoming unresponsive (*n* = 13), self-reported difficulty with the protocol (*n* = 3), scheduling conflicts (*n* = 3), or adverse events (*n* = 3, reported as moderate). This left a final analyzed sample size of 43 participants, including 22 assigned to the HFT group and 21 assigned to CON (Figure 2). The descriptive characteristics of the study population are noted in Table 1a (study completers) and Table 1b (total randomized subjects). The record of adverse events reflected no serious adverse events from any intervention in this study. There were, however, three moderately severe adverse events within the intervention group that led to discontinuation of study procedures. One subject in the CON group experienced right knee pain that was unrelated to the study protocol, which was resolved. One subject in the HFT group reported increased ankle instability after starting the program. The second subject reported muscle soreness and more ankle instability. And a third subject reported knee pain. Subjects did not report these adverse events during the trainer-supervised HFT sessions, but rather reported them during independent practice. All three subjects discontinued the protocol upon reporting adverse events. Although the absolute number is small, the concentration of events in unsupervised sessions suggests that supervision may be relevant to safe delivery. The overall elevated attrition rate observed in the HFT group is likely attributable to the demanding nature of the intervention, which required consistent weekly engagement across live virtual sessions and independent practice, representing a substantially greater time and behavioral commitment than the passive CON condition. Baseline outcome measures of HFT completers and non-completers are compared in Supplementary Table S3. Standardized mean differences were small across most measures, including the primary outcome of CAIT (SMD 0.23) and both co-secondary jump measures (jump height SMD −0.08; flight time SMD −0.10). The only measure exceeding 0.50 was FAAM ADL Global, on which completers scored higher at the baseline (SMD 0.71). The single CON non-completer precludes the equivalent comparison in that arm.

### 2.3. Cumberland Ankle Instability Tool

Ankle instability was assessed using the CAIT, a validated self-report, 9-item questionnaire used to assess functional ankle instability, in which each item is scored on a scale of 0–3, 0–4, or 0–5, yielding a total score ranging from 0 to 30, with higher scores reflecting greater ankle stability [24]. Study enrollment required a CAIT score of 25 or lower in at least one ankle (baseline), consistent with the established cutoff for functional ankle instability, which has demonstrated a sensitivity of 96.6% and specificity of 86.8% in discriminating between individuals with and without chronic ankle instability [25]. Scores were reported based on the worst-affected ankle (index ankle), defined as the ankle with the lowest reported score regardless of laterality, and improvements over time were interpreted as decreased ankle instability (or greater stability). The index ankle was identified at baseline and retained throughout all follow-up visits, and used as the outcome measure.

### 2.4. Foot and Ankle Ability Measure (FAAM)

Subjective self-reported ankle function was evaluated using the FAAM, which consists of two subscales: Activities of Daily Living (ADL; 21 items) and Sports (8 items). Each item is scored from 0 (unable to perform) to 4 (no difficulty), with subscale scores converted to a percentage of the maximum possible score for each domain, where higher values indicate greater functional capacity [26]. A Single-Item Global Score was also collected for both subscales, asking participants to rate their current level of function from 0 (inability to perform any usual daily activities) to 100 (ability to function at a level equivalent to before their foot or ankle issue) relative to their history with ankle problems. Improvements in subscale scores were interpreted as reductions in ankle-related functional limitations.

### 2.5. Maximal Countermovement Jump (CMJ)

Lower limb explosive power and neuromuscular function were assessed via maximal countermovement jump (CMJ) performance using the My Jump Lab application for iOS devices (My Jump Lab, Version 3, Carlos Balsalobre, Madrid, Spain). This application has been validated against ground reaction force platform measurements using a computation method, which was substantiated in clinical trials designed to estimate force, velocity, and power generated during countermovement jumps. The MJL has more than 30 tests that perform markerless motion tracking and have demonstrated high associations (r > 0.91) for vertical jump performance. Previous studies have shown that compared to a force platform, MJL has a mean difference of 2.0 ± 1.3 cm when algorithmically estimating CMJ height in healthy populations [27]. Outcome metrics included jump height (cm), propulsive force (N), relative propulsive force (N/kg), propulsive power (W), relative propulsive power (W/kg), mean velocity (m/s), take-off velocity (m/s), impulse (kg·m/s), and flight time (ms). Jump height and flight time are derived directly from markerless motion tracking of the recorded video, whereas force, relative force, power, relative power, mean velocity, take-off velocity, and impulse are algorithmically estimated from jump height and participant anthropometrics. Since several of these are interdependent, it was decided to use jump height and flight time as the co-secondary outcome measures and the others as exploratory algorithmic derivatives.

Eligibility required participants to have an iPhone XR (2018) or later model and also a minimum testing space of 10 ft × 10 ft with adequate ceiling clearance, partially standardizing device requirements and the testing environment. In recording jumping performance, we controlled within subjects to use the same device, camera frame rate, ambient lighting, and footwear from jump to jump. Prior to testing, participants were instructed to abstain from high-intensity exercise within the prior 72 h, and then were instructed to complete baseline, Week 8, and Week 12 assessments at approximately the same time of day at each timepoint. Technology requirements, including participants’ ability to access and operate the My Jump Lab application on their own device, were verified during the screening video call, at which point study staff also reviewed app instructions in detail. Participants began by downloading version 3 of the MJL app from the app store, then after creating an account, they were instructed to enter their body measurements, including weight, height, leg length, and hip height at a 90-degrees squat position. Participants adjusted the app setting to automatic collection and completed a standardized warm-up (two sets of 10 bodyweight squats with ~30 s rest between sets), before performing the biomechanical CMJ assessment. After device setup, participants positioned their phone at approximately waist height, angled to capture the full body from head to foot in the frontal plane. Participants centered themselves in the frame, held their hands on their hips throughout the jump, and remained stationary for approximately two seconds after entering the frame to allow the detection system to initialize. Once recording began, they executed the jump, allowing the app’s algorithm to analyze performance metrics. Participants performed up to three maximal CMJs, and the average across all trials was used for analysis. Upon completion, results were emailed to study staff.

### 2.6. Short-Form Health Survey 12 (SF-12)

SF-12 is a widely used 12-item questionnaire for evaluating general health and quality of life, assessing both physical and mental health domains. The questionnaire is structured across eight domains of well-being: physical functioning, role limitations due to physical health, role limitations due to emotional problems, energy or fatigue, emotional well-being, social functioning, pain, and general health [28]. Responses are recorded on scales ranging from 1 to 5 or 1 to 6 and are subsequently transformed to a standardized 0–100 scoring system, where higher scores indicate better overall well-being.

### 2.7. Statistical Analysis

Prior to inferential testing, data were screened for outliers. Outliers were identified through visual inspection of box plots and confirmed quantitatively by examination of studentized residuals, with values exceeding ±3 SD winsorized to that boundary within each group and time cell. This affected 32 of the analyzed observations: 13 countermovement jump values, 14 SF-12 values, and 5 FAAM values. No CAIT value required adjustment.

Following data screening, dependent variables were analyzed using linear mixed models fitted by the restricted maximum likelihood, with fixed effects for group, time, and the group × time interaction. Within-participant correlation was modeled using a random intercept for each participant. Time was entered as a categorical factor with three levels, and denominator degrees of freedom were obtained using the Satterthwaite approximation. Models were fitted to all available observations. Estimated marginal means were derived from each fitted model, and prespecified between- and within-group contrasts were evaluated with Holm–Bonferroni adjustment applied within contrast families, as well as 95% confidence intervals. Effect sizes were calculated as Cohen’s d, defined as the estimated marginal mean contrast divided by the model-implied marginal standard deviation, and interpreted as trivial (0–0.2), small (0.2–0.5), medium (0.5–0.8), or large (≥0.8). Because 23 randomized participants provided no post-randomization outcome data, a sensitivity analysis was conducted under a missing-not-at-random assumption for CAIT, vertical jump height, and flight time. A delta-adjusted pattern-mixture approach was used, in which the 22 HFT participants without follow-up data were assumed to have improved by a fixed amount (delta) less than observed HFT participants. The single CON participant without follow-up data was assumed to have changed by the observed CON mean, as informative dropout was a concern only in the intervention arm. Delta was increased incrementally to identify the tipping point at which each between-group difference in change ceased to reach significance.

Categorical outcomes were analyzed using chi-square tests with Cramér’s V for effect sizes, interpreted as small (0.07–0.21), medium (0.21–0.35), and large (0.35+). Because within-group comparisons of classification involved repeated binary measurements on the same participants, these were assessed using exact McNemar tests rather than chi-square. The responder status against baseline was not tested where it is fixed at zero by definition. Chi-square tests were retained for between-group comparisons at follow-up, which involve independent samples, and these comparisons are reported as exploratory. The chi-square test compares a test statistic to a continuous distribution, an approximation that holds when expected cell counts are large but degrades when they are small. Following the conventional threshold of five, Fisher’s exact test was performed in place of the chi-square approximation wherever an expected cell count fell below this value. Additionally, an exploratory two-tailed Pearson correlation analysis was conducted to examine the relationship between improvements in CAIT scores and CMJ performance, with r values interpreted as negligible (0–0.2), weak (0.2–0.4), moderate (0.4–0.6), strong (0.6–0.8), or very strong (≥0.8). Week 12 was designated the primary assessment timepoint, and the between-group difference in change from baseline to Week 12 was the primary treatment contrast. Statistical significance was set a priori at *p* < 0.05 for all analyses. All statistical procedures were performed using JASP (version 0.95.4, Amsterdam, The Netherlands). Figures were generated using Matplotlib (version 3.10.8, Chicago, IL, USA) in Python (version 3.12.3, Amsterdam, The Netherlands).

## 3. Results

### 3.1. CAIT Scores

**Figure 3:**
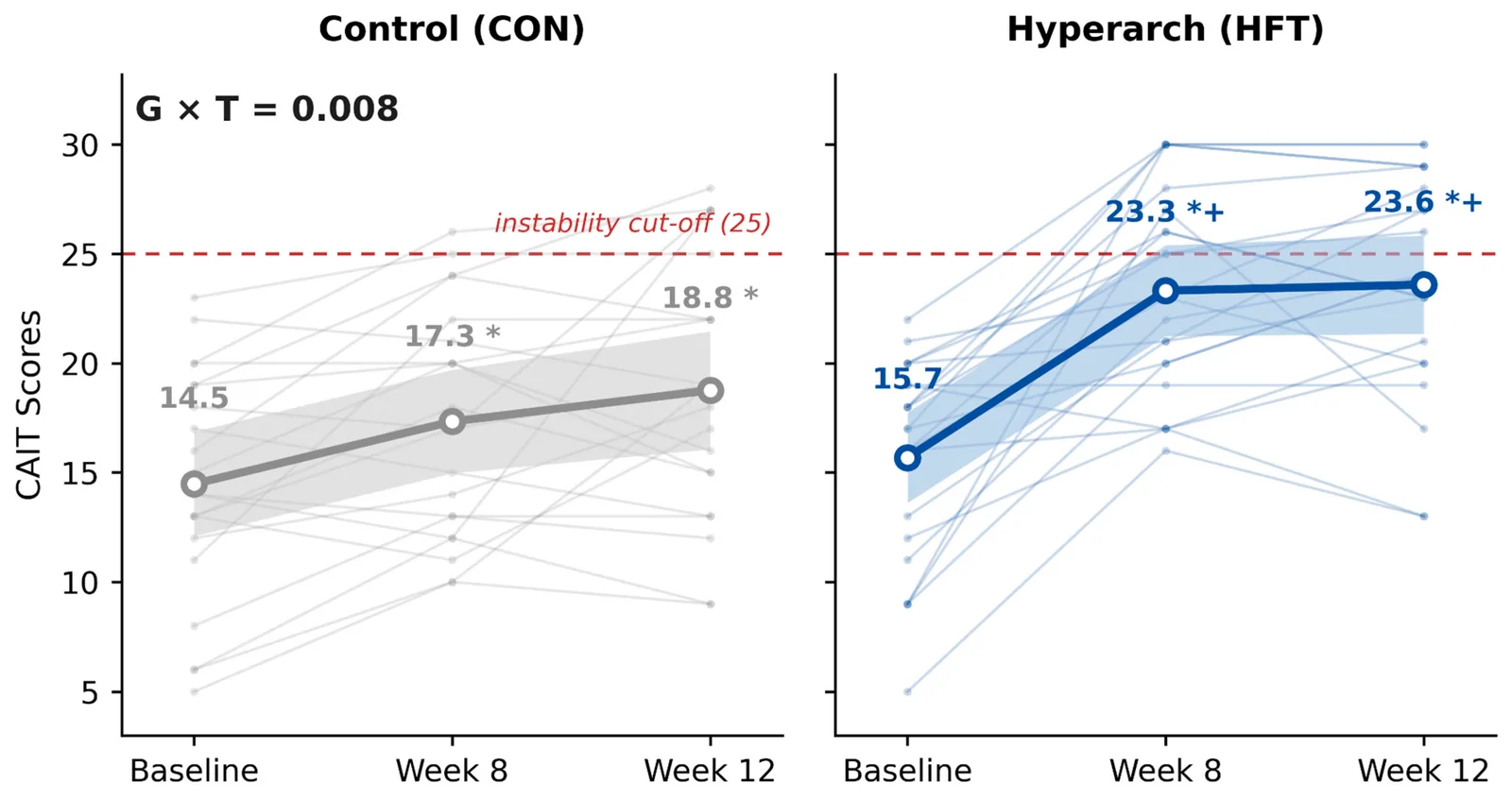
Cumberland Ankle Instability Tool (CAIT) scores (CON *n* = 21; HFT *n* = 22). Data are shown in grey for CON (**left panel**) and blue for HFT (**right panel**). Thin lines show individual participant trajectories, drawn between available timepoints only for completer subjects. Bold lines show group means with shaded 95% confidence intervals. The dashed red line indicates the instability cut-off of 25. G × T = group × time interaction (*p* = 0.008). * Significant (*p* < 0.05) within-group difference from baseline. + Significant (*p* < 0.05) between-group difference at that timepoint.

The primary outcome variable, CAIT scores, is shown in Table 2 and Figure 3. Both groups improved over time (CON Baseline–Week 8: *p* = 0.050, d = 0.56; Baseline–Week 12: *p* = 0.002, d = 0.83) (HFT Baseline–Week 8: *p* < 0.001, d = 1.49; Baseline–Week 12: *p* < 0.001, d = 1.54). There was a significant group × time interaction in CAIT scores, F(2, 82) = 5.10, *p* = 0.008, with the HFT group scoring higher than CON at Week 8 (5.99 points, 95% CI 2.86 to 9.11, d = 1.16, *p* = 0.002) and at Week 12 (4.83 points, 95% CI 1.71 to 7.95, d = 0.94, *p* = 0.014). No significant change occurred in the HFT group between Week 8 and Week 12 (*p* = 0.889). The between-group difference in change from baseline favored HFT by 4.78 points at Week 8 (95% CI 1.67 to 7.89, d = 0.93, *p* = 0.006) and by 3.62 points at Week 12 (95% CI 0.52 to 6.73, d = 0.70, *p* = 0.023). Additionally, a delta-adjusted sensitivity analysis was conducted for the primary contrast to the unobserved HFT participants. For CAIT, the between-group difference in change to Week 12 had a tipping point delta of 0.76 points, equivalent to a mean change of 7.15 points in unobserved participants, against 7.91 that we actually observed. The corresponding tipping point at Week 8 was 4.92 points, equivalent to a mean change of 2.72 points in unobserved participants, against 7.64 that we actually observed.

Categorical CAIT outcomes are presented in Table 3. Within-group changes in classification were assessed using exact McNemar tests. In the HFT group, the proportion scoring at or below the instability cut-off of 25 fell from 100% at baseline to 63.6% at Week 8 (*p* = 0.008) and 59.1% at Week 12 (*p* = 0.004), with no further change between Week 8 and Week 12 (*p* = 1.000). In the CON group, the corresponding proportions were 95.2% at Week 8 and 81.0% at Week 12, neither of which differed significantly from the baseline (*p* = 1.000 and *p* = 0.125). At Week 12, 40.9% of HFT participants scored above the cut-off compared with 19.0% of CON. For the cut-off classification at Week 8, the minimum expected cell count was 4.40, so Fisher’s exact test was used for that comparison rather than chi-square, which was significant (*p* = 0.021, V = 0.388); the comparison at Week 12 was analyzed by chi-square and was not significant (*p* = 0.119, V = 0.238). At Week 12, 81.8% of HFT and 52.4% of CON participants achieved an improvement of at least 3 points, the minimal clinically important difference (MCID) for this instrument, which was significantly different between the groups (*p* = 0.039, V = 0.314). The responder status at baseline is zero by definition and was not tested. All categorical between-group comparisons are exploratory. Finally, the HFT group mean at Week 12 (23.59 ± 5.08) remained below the CAIT instability cut-off, indicating a reduction in perceived ankle instability rather than normalization.

### 3.2. FAAM Scores

The primary outcome variable, FAAM Survey scores, is shown in Table 2. No group × time interaction reached significance for any FAAM outcome—ADL Percent: F(2, 82) = 0.55, *p* = 0.579; ADL Global: F(2, 82) = 0.15, *p* = 0.858; Sport Percent: F(2, 82) = 2.77, *p* = 0.068; Sport Global: F(2, 82) = 2.89, *p* = 0.061. On post hoc analysis, the HFT group improved from baseline on all four scores: ADL Percent at Week 12 (95% CI 1.97 to 11.27, *p* = 0.049, d = 0.67), ADL Global at Week 8 (95% CI 1.42 to 9.67, *p* = 0.049, d = 0.72) and Week 12 (95% CI 2.92 to 11.17, *p* = 0.009, d = 0.91), Sport Percent at Week 8 and Week 12 (95% CI 6.27 to 17.36 and 9.23 to 20.32, both *p* < 0.001, d = 0.87 and 1.09), and Sport Global at Week 8 and Week 12 (95% CI 7.55 to 17.18 and 8.64 to 18.27, both *p* < 0.001, d = 1.12 and 1.22). The CON group improved on ADL Global at Week 12 (95% CI 1.55 to 9.99, *p* = 0.049, d = 0.75) but showed no other significant within-group change. Between groups, HFT scored higher than CON on ADL Percent at Week 8 (8.61 points, 95% CI 2.61 to 14.60, d = 0.87, *p* = 0.049), on ADL Global at Week 8 (6.80 points, 95% CI 2.09 to 11.50, d = 0.88, *p* = 0.041) and Week 12 (6.53 points, 95% CI 1.83 to 11.24, d = 0.84, *p* = 0.049), and on Sport Global at Week 12 (9.49 points, 95% CI 2.78 to 16.20, d = 0.86, *p* = 0.043). Sport Percent did not differ between groups at any visit. Interpretation of the FAAM is limited by ceiling compression, with 45.5% of the HFT group scoring the maximum of 100 on the ADL Percent scale at Week 12, which constrains the detectable between-group difference. Between-group differences in change from baseline did not reach significance for any FAAM outcome: ADL Percent 3.48 points at Week 8 (95% CI −3.18 to 10.13, d = 0.35, *p* = 0.603) and 1.36 points at Week 12 (95% CI −5.30 to 8.01, d = 0.14, *p* = 0.686); ADL Global 1.53 points at Week 8 (95% CI −4.36 to 7.43, d = 0.20, *p* = 1.000) and 1.27 points at Week 12 (95% CI −4.63 to 7.17, d = 0.16, *p* = 1.000); Sport Percent 8.21 points at Week 8 (95% CI 0.28 to 16.15, d = 0.61, *p* = 0.085) and 8.05 points at Week 12 (95% CI 0.11 to 15.99, d = 0.60, *p* = 0.085); and Sport Global 6.72 points at Week 8 (95% CI −0.17 to 13.61, d = 0.61, *p* = 0.061) and 7.62 points at Week 12 (95% CI 0.73 to 14.51, d = 0.69, *p* = 0.061).

### 3.3. Vertical Jump Performance

**Figure 4:**
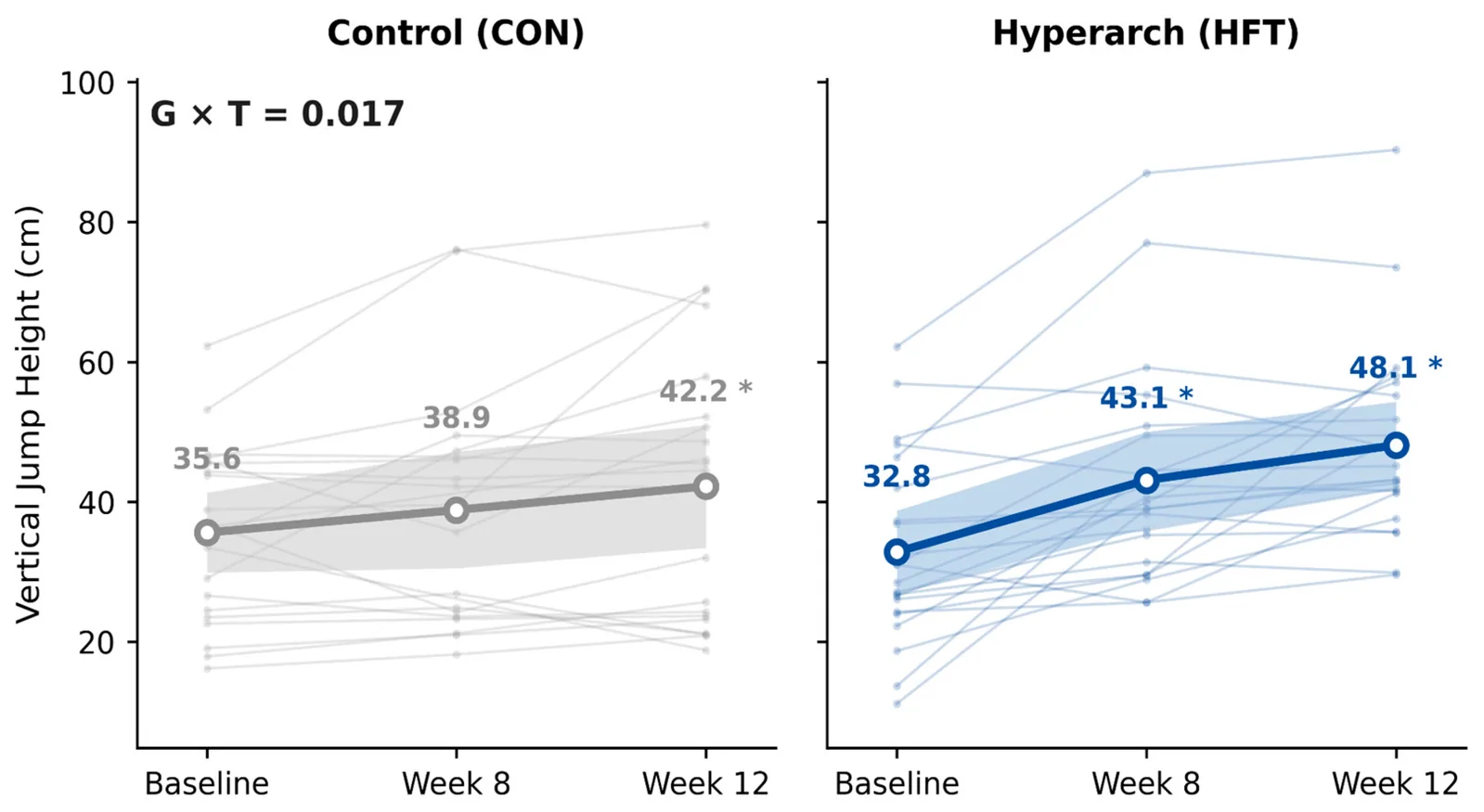
Vertical jump height (CON *n* = 21; HFT = 22). Data are shown in grey for CON (**left panel**) and blue for HFT (**right panel**). Thin lines show individual participant trajectories, drawn between available timepoints only for completer subjects. Bold lines show group means with shaded 95% confidence intervals. G × T = group × time interaction (*p* = 0.017). * Significant (*p* < 0.05) within-group differences from baseline.

All indices for the secondary outcome variable, CMJ performance, are shown in Table 4 and Figure 4. Significant group × time interactions were observed for the two co-secondary variables of Vertical Jump Height (F(2, 82) = 4.25, *p* = 0.017) and Flight Time (F(2, 82) = 5.58, *p* = 0.005), as well as two algorithmic derivatives (exploratory outcomes), Mean Velocity (F(2, 82) = 7.06, *p* = 0.001) and Take-off Velocity (F(2, 82) = 5.62, *p* = 0.008). No significant interaction was observed for Force (F(2, 82) = 0.45, *p* = 0.640), Relative Force (F(2, 82) = 0.91, *p* = 0.407), Power (F(2, 82) = 0.53, *p* = 0.590), Relative Power (F(2, 82) = 1.13, *p* = 0.327) or Impulse (F(2, 82) = 0.22, *p* = 0.806); these variables are estimated algorithmically rather than measured directly.

For the two co-secondary variables, HFT improved from baseline in Vertical Jump Height by 10.26 cm at Week 8 (95% CI 5.80 to 14.72, d = 0.67, *p* < 0.001) and 15.27 cm at Week 12 (95% CI 10.81 to 19.74, d = 0.99, *p* < 0.001), as well as Flight Time by 77.6 ms at Week 8 (95% CI 45.5 to 109.6, d = 0.72, *p* < 0.001) and 113.1 ms at Week 12 (95% CI 81.1 to 145.1, d = 1.05, *p* < 0.001). Significant improvements were also seen in the HFT group for algorithmic derivatives of Mean Velocity by 0.19 m/s at Week 8 (95% CI 0.11 to 0.27, d = 0.72, *p* < 0.001) and 0.31 m/s at Week 12 (95% CI 0.23 to 0.39, d = 1.19, *p* < 0.001); Take-off Velocity by 0.38 m/s at Week 8 (95% CI 0.23 to 0.54, d = 0.72, *p* < 0.001) and 0.56 m/s at Week 12 (95% CI 0.40 to 0.71, d = 1.05, *p* < 0.001); Power by 2745 W at Week 12 (95% CI 1452 to 4038, d = 0.54, *p* < 0.001); and Impulse by 38.1 N·s at Week 12 (95% CI 17.4 to 58.8, d = 0.46, *p* = 0.004). The CON group also improved in Vertical Jump Height by 6.61 cm at Week 12 (95% CI 2.04 to 11.18, d = 0.43, *p* = 0.036), and Impulse by 33.8 N·s at Week 12 (95% CI 12.7 to 55.0, d = 0.40, *p* = 0.017). The CON group, which received no intervention, increased mean jump height by 18.6% over the 12 weeks (HFT by comparison increased by 46.49%), indicating that familiarization may have partially contributed to the change observed in both groups.

Between-group differences in change from baseline favored HFT for Vertical Jump Height by 7.50 cm at Week 8 (95% CI 0.99 to 14.01, d = 0.49, *p* = 0.024) and 8.66 cm at Week 12 (95% CI 2.27 to 15.05, d = 0.56, *p* = 0.017), and for Flight Time by 60.1 ms at Week 8 (95% CI 13.4 to 106.8, d = 0.56, *p* = 0.012) and 72.0 ms at Week 12 (95% CI 26.2 to 117.8, d = 0.67, *p* = 0.005). Corresponding differences for Mean Velocity were 0.147 m/s at Week 8 (95% CI 0.030 to 0.264, d = 0.56, *p* = 0.014) and 0.211 m/s at Week 12 (95% CI 0.097 to 0.326, d = 0.80, *p* < 0.001). For Take-off Velocity, they were 0.296 m/s at Week 8 (95% CI 0.067 to 0.525, d = 0.56, *p* = 0.012) and 0.354 m/s at Week 12 (95% CI 0.129 to 0.578, d = 0.67, *p* = 0.005).

Additionally, a sensitivity analysis was applied to the co-secondary jump variables to the unobserved HFT participants. For vertical jump height, the tipping point was 4.21 cm at Week 8, equivalent to a mean change of 6.05 cm in unobserved participants against 10.26 cm that we actually observed, and 4.73 cm at Week 12, equivalent to a mean change of 10.54 cm against 15.27 cm observed. For flight time, the tipping point was 41.3 ms at Week 8, equivalent to a mean change of 36.2 ms in unobserved participants against 77.6 ms observed, and 55.2 ms at Week 12, equivalent to 57.9 ms in unobserved participants against 113.1 ms observed.

For several of the jump performance variables, particularly power and force, the standard deviations approached the group means, reflecting wide between-participant dispersion in estimated kinetic output. Internal consistency of the derived metrics was verified, with jump height reproducing from flight time and independently from take-off velocity at r greater than 0.999, and with estimated power equaling the product of estimated force and mean velocity; however, absolute calibration could not be verified against a force platform, and these variables are interpreted as relative change within participants rather than as absolute physiological quantities.

### 3.4. SF-12 Outcomes

The exploratory outcome variable, SF-12 survey results, is shown in Table 5 and reported as exploratory. There were significant Group × Time interactions reached for Physical Function (*p* = 0.045), Limits due to Physical Health (*p* = 0.030) and General Health (*p* = 0.043), with CON tending to decrease over time and HFT to increase. A main effect for group was also observed for Physical Function (F(1, 41) = 8.82, *p* = 0.005) and Perceived Pain (F(1, 41) = 7.17, *p* = 0.011). On post hoc analysis, the HFT group scored higher than CON on Physical Function at Week 8 (14.7 points, 95% CI 6.1 to 23.3, d = 1.04, *p* = 0.009) and on Perceived Pain at Week 12 (13.2 points, 95% CI 4.0 to 22.4, d = 0.88, *p* = 0.047). No other between-group or within-group contrast reached significance. Between-group differences in change from baseline favored HFT for Physical Function by 12.9 points at Week 8 (95% CI 2.5 to 23.3, d = 0.92, *p* = 0.031), for Limits due to Physical Health by 26.1 points at Week 8 (95% CI 6.3 to 45.9, d = 1.11, *p* = 0.021), and for General Health by 10.4 points at Week 12 (95% CI 2.2 to 18.7, d = 0.70, *p* = 0.028); the remaining contrasts did not reach significance.

## 4. Discussion

In this study, it was found that 12 weeks of a multicomponent training program emphasizing fascia-targeted techniques led to improved perceived ankle stability and function, as well as increases in objective indices of physical performance through the vertical jump performance. Importantly, these improvements were made in lieu of traditional exercise and strength training practices, including the absence of hip hinge or other triple extension movements, such as squats or deadlifts, as well as novel barefoot training exercises by removal of external shoe support.

A primary component of fascia training requires that minimal tissue damage and resultant inflammation occur, as chronic inflammation may result in additional fascia tissue damage [29,30], so avoiding these types of loaded movements is a key detail in fascia-oriented programs. Due to the fact that fascia remodeling likely requires a lower-intensity exercise stimulus [16,17], it is theoretically plausible (though speculative) that multi-component programs emphasizing fascia training techniques, such as HFT, are able to achieve a mechanical threshold that results in fascia remodeling independent of muscle protein synthesis or skeletal muscle remodeling. Mechanical adaptation of the fascial system could, in theory, affect joint stability and movement performance through hypothesized enhancements in fascial stiffness, elasticity, and force transmission [11]. Moreover, targeting the myofascia in the foot–ankle support system may have unique upstream benefits for lower body kinetic chaining. Fascial tissue is rife with proprioceptors, which provide feedback to the neuromuscular system in regard to joint angles and positions in space [31]. This proprioceptive feedback is a key mechanism in the efferent/afferent signaling pathways between the brain and peripheral tissue. Each subsequent command sent by the motor cortex is heavily influenced by the previous afferent feedback from the peripheral tissue [32], and this feedback loop helps create the basis for continual human movement. Interestingly, these enhanced feedback mechanisms may be augmented through the employment of barefoot training. While this theory is plausible, it remains speculative and must be further substantiated by future research.

By removing external shoe support and repeatedly loading the foot–Achilles system through fascial aponeurosis, barefoot exercise may challenge the connective tissues responsible for stiffness regulation (such as myofibroblast cells), elastic energy storage, recoil, and force transmission. Barefoot practice is specifically used to enhance proprioceptive feedback, and recent studies have suggested that barefoot exercise can improve balance and stability through enhanced proprioception [33]. The plantar structures of the foot play a critical role in sensing ground reaction forces and contributing to postural stability, balance, and lower limb coordination. Through both mechanical and neuromuscular pathways, improvements in foot function may influence proximal muscle recruitment patterns [34]. It is plausible that enhanced sensory feedback from the foot may improve motor unit recruitment, timing, and coordination throughout the lower limb, leading to improved synchronization between distal and proximal segments. This, in turn, hypothetically, could contribute to greater force production during the propulsion phase of a countermovement jump [35]. Indeed, this hypothesis is consistent with the work of Yamauchi and Koyama [36]. They tested 31 healthy young men and found that toe-flexor strength and especially toe-flexor rate of force development (RFD) were positively correlated with jump performance. For countermovement jump height, toe-flexor RFD correlated at r = 0.548; and normalized maximal toe-flexor strength correlated at r = 0.444. The relationships were also significant for squat jumps and rebound jumps.

That said, it must be acknowledged that other activities, including toe raises, plyometrics, and various balance exercises, were also performed. Therefore, the absence of more traditional movements does not establish that the observed adaptations occurred solely through a fascia-specific pathway. These exercises can also engage conventional neuromuscular pathways and contribute to the observed adaptations. Thus, while fascial adaptation is a highly plausible mechanistic contributor, it cannot be isolated as the sole mechanism and remains speculative. Rather, the observed effects likely reflect a combination of fascial, neuromuscular, and other physiological adaptations.

As previously mentioned, prior studies have suggested that fascial treatment and training can acutely improve aspects of standing balance [13,22], and the results of this study also showed a multicomponent intervention emphasizing fascial training techniques led to increased CAIT scores (the primary outcome variable), which reflect improvements in perceived ankle stability. It is also well established that force production and the vertical jump performance are reliant on stability [37,38], and enhancing the stability of the lower limb support system through fascial training is hypothetically a significant factor that led to the improvements in CMJ performance shown in this study (the secondary outcome variable). Indeed, through additional exploratory analysis, we found a moderate correlation (r = 0.4291, 95% CI 0.009 to 0.720, two-tailed *p* = 0.046) between improvements in CMJ height and CAIT scores, which suggests that there is a positive relationship between improving subjective ankle stability and increasing CMJ performance. Future research should further investigate the foot-to-glute connection in relation to jumping performance.

The multicomponent HFT program examined in this study also utilized mental imagery practices to promote enhanced kinetic chaining [39]. Mental imagery is a sport psychology technique, which has potentially positive effects on performance, combined with minimal fatigue and almost non-existent injury risk [40,41]. Currently, it is unclear of the exact physiological or psychological mechanism that links performance improvements with mental imagery. Regardless of the mechanism, the use of mental imagery, and the emphasis on the foot–ankle–glute connection in the Hyperarch fascia training program, may provide a unique advantage in regard to the kinetic chain of the ankle, knee, and hip, although the level of contribution and the extent of involvement is unknown.

Subjective health and lifestyle surveys utilizing SF-12 (the exploratory outcome variable) were also examined as an exploratory indication of general perceived health and quality of life. The primary group-by-time interactions observed were for the following categories: Perceived Physical Function, Limits due to Physical Health, and General Health, as well as an overall group difference for Pain, with the HFT group tending to produce better results overall, particularly with significantly higher scores than CON for Physical Function at Week 8 and Perceived Pain at Week 12. Additionally, we further examined subjective ankle health-specific surveys using the Foot and Ankle Ability Measure (FAAM) (the primary outcome variable), directly assessing ankle instability and its effects on daily living, which likely provides a more sensitive measurement in the context of this study. It was found that no group × time interaction reached significance for any FAAM outcome. However, the HFT group showed significant within-group improvements in FAAM scores relative to the CON group, including ADL percent and Global at Week 8 and Sport Global at Week 12. In turn, an improvement in subjective ankle function may hypothetically have led to increased overall confidence and self-efficacy within sporting tasks [42]. These differences may possibly explain at least some of the improvement in vertical jump performance shown in the HFT group. Importantly, interpretation of the FAAM is limited by ceiling compression, with 45.5% of the HFT group scoring the maximum of 100 on the ADL Percent scale at Week 12, which constrains the detectable between-group difference.

In terms of practical application, it is important to note that the vast majority of muscular strain-type injuries actually occur in either the myofascia or musculotendinous junction [43], which represents an intriguing rehabilitative advantage for fascia training. Fascial anatomy provides a theoretical framework for understanding these effects. The plantar fascia, achilles tendon, calf complex, hamstrings, sacrotuberous ligament, thoracolumbar fascia, and gluteal muscles are interconnected through continuous myofascial structures often described as the posterior chain. Improved stiffness, elasticity, and force transfer within these tissues may enhance the efficiency of energy storage and release during explosive movements [44]. Enhanced sensory feedback from the foot may improve motor unit recruitment, timing, and coordination throughout the lower limb. Thus, hypothetically, strengthening of these anatomical features may provide a significant underpinning of injury rehabilitation.

### Limitations

As with most exercise-related research, a primary limitation of this study is the overall sample size of *n* = 21 in the CON group and *n* = 22 in the HFT group. However, these group sizes are actually larger than previously reported average exercise-related study sample sizes (19 ± 11) [45], so while this may be a limitation of this study, it is certainly in line with the field average. Additionally, the outcomes of this study are limited to the examined population of physically active adults. Future research should investigate the effects of fascia training on both younger and older individuals to better understand training outcomes.

Another limitation is that eligibility required a CAIT score of 25 or lower, and selecting participants on the basis of a low baseline value maximizes the expected magnitude of regression to the mean at follow-up. The CON group, which received no intervention, nonetheless improved significantly over twelve weeks in both CAIT scores and vertical jump performance, which is consistent with a combination of either regression to the mean, repeated questionnaire exposure, familiarization or Hawthorne effects. However, these processes operate in both arms, and therefore, cannot fully account for the greater performance results seen in HFT for CAIT and vertical jump.

Since the HFT program is multicomponent, combining myofascial rolling, foot-intrinsic strengthening, single-leg balance work, plyometric activities, movement drills, barefoot practice and mental imagery, each of these has plausible independent effects on neuromuscular function, and the present design cannot apportion the observed effects among them.

One principal limitation of this trial is the rates of attrition. Of the 66 participants randomized at a ~2:1 ratio to HFT (44) and CON (22), 23 contributed no post-randomization outcome data and were excluded from analysis. Importantly, withdrawal was markedly differential between groups (HFT 50.0%, CON 4.5%). The delta-adjusted sensitivity analysis is therefore limited by the proportion of unobserved participants, as 22 of 44 HFT participants contributed no follow-up data. After reviewing individual withdrawal notes, HFT attrition was attributable mainly to loss of contact, the physical demands and time commitment of the protocol, or scheduling conflict, whereas CON attrition was driven entirely by loss of contact. The overall elevated attrition rate observed in the HFT group is likely attributable to the demanding nature of the intervention, which required consistent weekly engagement across live virtual sessions and independent practice, representing a substantially greater time and behavioral commitment than the passive CON condition. As a consequence of the greater commitment, the analyzed HFT group likely reflects participants who were comparatively more adherent, tolerant, and motivated about the intervention than the full allocated cohort. Because the participants who remained were likely more motivated, the reported effects may overstate what would be expected in the full randomized cohort. The reported results should be interpreted with this specificity toward engaged participants in mind. Completed subjects complied with attending all sessions in HFT; however, the quantitative training volume was not monitored, as it was individualized and varied based on the subjects’ progression in the program, which is a limitation of this report.

The lack of consensus regarding fascia-specific training parameters remains a limitation of the literature. Unlike strength and endurance training, standardized recommendations for frequency, intensity, and progression have not been established. Future studies should define these parameters and directly assess fascial properties using shear wave elastography or an equivalent validated method.

The passive control condition also limits mechanistic interpretation, as groups differ in physical activity, exercise exposure, supervision, attention, and expectation of benefit. Future studies should use a volume-matched active comparator matched for training volume, supervision, and contact time, differing primarily in components theorized to target fascia.

Final limitations include that the participants and coaches were not blinded, outcomes were self-reported, and the HFT group received weekly coaching, making expectancy effects difficult to separate from treatment effects. Lastly, the absence of post-intervention, long-duration follow-up prevents the assessment of the durability of observed benefits.

## 5. Conclusions

The findings of this randomized controlled trial suggest that a multicomponent training program emphasizing fascia-targeted techniques led to improved perceived ankle stability and function, as well as increases in objective indices of physical performance through vertical jump performance. Future studies incorporating electromyography, force plate analysis, ultrasound assessment of fascial structures, and measures of rate of force development may help clarify the relative contribution of each of these possible theoretical mechanisms.

## Figures and Tables

**Table 1:** (**a**) Study completers only: Baseline descriptive characteristics. (**b**) All randomized subjects: Baseline descriptive characteristics.

(**a**)				
	**Age (year)**	**Height (m)**	**Weight (kg)**	**BMI (kg/m^2^)**
Control	35.24 ± 7.02	1.73 ± 0.11	83.86 ± 19.34	27.77 ± 4.66
Males (*n* = 10)				
Females (*n* = 11)				
Total (*n* = 21)				
HFT	31.86 ± 5.57	1.74 ± 0.12	76.97 ± 17.22	25.13 ± 3.43
Males (*n* = 14)				
Females (*n* = 8)				
Total (*n* = 22)				
SMD	0.53	−0.097	0.37	0.64
(**b**)				
	**Age (year)**	**Height (m)**	**Weight (kg)**	**BMI (kg/m^2^)**
Control	34.9 ± 7.1	1.7 ± 0.1	84.1 ± 18.9	27.6 ± 4.6
Males (*n* = 11)				
Females (*n* = 11)				
Total (*n* = 22)				
HFT	31.1 ± 5.9	1.7 ± 0.1	74.7 ± 14.5	24.6 ± 3.0
Males (*n* = 29)				
Females (*n* = 15)				
Total (*n* = 44)				
SMD	0.58	−0.01	0.55	0.79

Data are means (standard deviations). BMI = body mass index. SMD = Standardized Mean Differences.

**Table 2:** Ankle health scores.

		Baseline	Week 8	Week 12	G × T
CAIT Score	CON	14.48 ± 5.26	17.33 ± 5.19 *	18.76 ± 5.92 *	*p* = 0.008
	HFT	15.68 ± 4.66	23.32 ± 4.68 *#+^	23.59 ± 5.08 *#+^	
FAAM ADL		Baseline	Week 8	Week 12	G × T
Percent Score	CON	85.60 ± 18.57	88.66 ± 11.73	91.55 ± 11.53	*p* = 0.579
	HFT	91.16 ± 8.27	97.27 ± 3.52 +	98.04 ± 2.56 *	
Global Score	CON	81.40 ± 10.04	85.48 ± 7.17	87.24 ± 6.93 *	*p* = 0.858
	HFT	86.73 ± 9.21	92.27 ± 6.86 *#+^	93.77 ± 5.73 *#+^	
FAAM Sport		Baseline	Week 8	Week 12	G × T
Percent Score	CON	75.80 ± 17.47	79.61 ± 14.24	82.74 ± 14.31	*p* = 0.068
	HFT	76.99 ± 14.09	88.78 ± 11.72 *	91.76 ± 9.07 *	
Global Score	CON	75.40 ± 11.65	81.05 ± 10.49	81.24 ± 12.63	*p* = 0.061
	HFT	77.27 ± 14.43	89.64 ± 7.83 *#	90.73 ± 7.79 *#+	

Data are mean ± standard deviations. CON, control, HFT, hyperarch. Cumberland Ankle Instability Tool (CAIT). Foot and Ankle Ability Measure (FAAM), Activities of Daily Living (ADL). G × T = group × time interactions. * significant (*p* < 0.05) within-group difference from baseline. (#) = significantly different between groups (*p* < 0.05) compared to baseline; (+) = significantly different between groups (*p* < 0.05) compared to week 8; (^) = significantly different between groups (*p* < 0.05) compared to week 12.

**Table 3:** CAIT scores at or below instability threshold and MCID scores.

Classification	Timepoint	CON (*n* = 21)	CON vs. Baseline *p*	HFT (*n* = 22)	HFT vs. Baseline *p*	Between Groups *p* (V)
Cut-off (score ≤ 25)	Baseline	21/21 (100.0%)	—	22/22 (100.0%)	—	—
	Week 8	20/21 (95.2%)	1.000	14/22 (63.6%)	0.008	0.021 (0.388) ^a^
	Week 12	17/21 (81.0%)	0.125	13/22 (59.1%)	0.004	0.119 (0.238)
	Week 8 vs. Week 12	—	0.250	—	1.000	—
MCID (≥3-point improvement)	Baseline	0/21 (0.0%)	—	0/22 (0.0%)	—	—
	Week 8	11/21 (52.4%)	—	17/22 (77.3%)	—	0.087 (0.261)
	Week 12	11/21 (52.4%)	—	18/22 (81.8%)	—	0.039 (0.314)
	Week 8 vs. Week 12	—	1.000 (0.000)	—	1.000	—

Values are the number and percentage of participants at or below the instability cut-off of 25, and the number and percentage achieving the minimal clinically important difference (MCID) of 3 points from baseline. Within-group comparisons against baseline are exact McNemar tests. Between-group comparisons at each follow-up timepoint are chi-square tests with Cramér’s V and are exploratory. ^a^ Analyzed by Fisher’s exact test, as the minimum expected cell count was 4.40. Em dashes indicate comparisons that were not tested: baseline between-group comparisons, where all participants met the cut-off, and MCID comparisons against baseline, where the responder status is zero by definition.

**Table 4:** Vertical jump performance.

Vertical Jump Performance		Baseline	Week 8	Week 12	G × T
Jump Height (cm)	CON	35.62 ± 12.55	38.85 ± 17.27	42.23 ± 19.28 *	*p* = 0.017
	HFT	32.85 ± 13.31	43.11 ± 15.57 *	48.12 ± 13.98 *	
Force (N)	CON	3451.66 ± 2417.19	4096.72 ± 3249.05	4236.39 ± 3501.21	*p* = 0.640
	HFT	3111.62 ± 2040.30	3639.33 ± 2539.17	4248.26 ± 3358.96 *	
Relative Force (N/kg)	CON	37.75 ± 24.83	43.81 ± 33.11	46.09 ± 36.23	*p* = 0.407
	HFT	41.24 ± 27.50	47.55 ± 32.25	56.17 ± 43.84 *	
Power (W)	CON	4423.95 ± 3215.39	5671.22 ± 5422.26	6209.95 ± 5976.93	*p* = 0.590
	HFT	4173.43 ± 3498.83	5649.94 ± 5134.55	6918.18 ± 6410.41 *	
Relative Power (W/kg)	CON	49.34 ± 34.77	61.10 ± 55.78	67.92 ± 62.49	*p* = 0.327
	HFT	54.68 ± 45.41	72.35 ± 60.56	90.63 ± 81.51 *	
Mean Velocity (m/s)	CON	1.30 ± 0.24	1.35 ± 0.30	1.40 ± 0.33	*p* = 0.001
	HFT	1.24 ± 0.26	1.43 ± 0.24 *	1.56 ± 0.20 *#	
Take-off Velocity (m/s)	CON	2.60 ± 0.48	2.70 ± 0.61	2.80 ± 0.66	*p* = 0.008
	HFT	2.49 ± 0.51	2.87 ± 0.49 *	3.04 ± 0.42 *	
Impulse (kg m/s)	CON	240.97 ± 79.05	272.18 ± 104.26	274.81 ± 115.39	*p* = 0.806
	HFT	203.94 ± 59.74	222.76 ± 69.99	242.07 ± 63.45 *	
Flight Time (ms)	CON	530.67 ± 96.91	551.16 ± 122.86	571.76 ± 134.78	*p* = 0.005
	HFT	507.27 ± 104.87	584.84 ± 99.67 *	620.36 ± 85.95 *	

Data are mean ± standard deviations. CON, control, HFT, hyperarch. cm (centimeters), N (newtons), kg (kilograms), W (watts), m/s (meters per second), ms (milliseconds). G × T = group × time interactions. * significant (*p* < 0.05) within-group difference from baseline. (#) = significantly different between groups (*p* < 0.05) compared to baseline.

**Table 5:** Short-Form Health Survey 12 (SF-12).

SF-12		Baseline	Week 8	Week 12	G × T
Physical Function	CON	94.05 ± 15.62	84.52 ± 20.12	85.71 ± 18.66	*p* = 0.045
	HFT	95.45 ± 12.53	98.86 ± 5.33 ^+^	96.59 ± 8.78	
Limits due to Physical Health	CON	90.48 ± 25.59	80.95 ± 37.00	98.33 ± 7.66	*p* = 0.030
	HFT	84.09 ± 35.81	100.00 ± 0.00	98.44 ± 7.30	
Limits due to Emotional Health	CON	85.71 ± 35.86	90.48 ± 25.59	92.86 ± 23.90	*p* = 0.108
	HFT	97.73 ± 10.66	93.18 ± 23.38	95.45 ± 21.32	
Energy/Fatigue	CON	66.67 ± 21.29	67.62 ± 19.47	68.57 ± 18.52	*p* = 0.750
	HFT	71.82 ± 13.32	72.73 ± 15.79	70.91 ± 16.01	
Emotional Well-being	CON	74.29 ± 21.35	77.62 ± 14.46	78.10 ± 16.01	*p* = 0.920
	HFT	78.64 ± 13.20	83.18 ± 13.59	81.82 ± 10.97	
Social Function	CON	90.48 ± 20.12	91.67 ± 12.08	91.67 ± 14.43	*p* = 0.947
	HFT	92.05 ± 11.92	94.32 ± 13.21	93.18 ± 11.40	
Pain	CON	79.76 ± 20.34	82.14 ± 14.02	81.12 ± 16.97	*p* = 0.291
	HFT	84.09 ± 14.53	90.91 ± 12.31	94.32 ± 10.72 ^	
General Health	CON	80.95 ± 10.91	77.38 ± 15.62	76.19 ± 14.74	*p* = 0.043
	HFT	79.55 ± 14.71	79.55 ± 18.32	85.23 ± 14.76	

Data are mean ± standard deviations. CON, control, HFT, hyperarch. G × T = group × time interactions. (+) = significantly different between groups (*p* < 0.05) compared to week 8. (^) = significantly different between groups (*p* < 0.05) compared to week 12.

## Data Availability

The original contributions presented in this study are included in the article and Supplementary Materials. Further inquiries can be directed to the corresponding author.

## Supplementary Materials

The following supporting information can be downloaded at https://www.mdpi.com/article/10.3390/sports14100468/s1, Table S1: CONSORT checklist; Table S2: Study eligibility criteria; Table S3: Baseline hyperarch fascia training completers versus non-completers; Table S4: Hyperarch fascia training protocol.

## Abbreviations

The following abbreviations are used in this manuscript:

ADL	Activities of Daily Living
CAI	Chronic Ankle Instability
CAIT	Cumberland Ankle Instability Tool
cm	Centimeters
CMJ	Countermovement Jump
CON	Control Group
FAAM	Foot and Ankle Ability Measure
HFT	Hyperarch Fascia Training
kg	Kilograms
MJL	My Jump Lab
ms	Milliseconds
m/s	Meters Per Second
N	Newtons
SF-12	Short Form Health Survey 12
W	Watts
