# Supplementary material for: The Impact of Hyperarch Fascia Training on Perceived Ankle Instability and Vertical Jump Performance in Physically Active Men and Women
Source: Sports (Basel). 2026 Oct 20;14(10):468. doi: 10.3390/sports14100468 (PMC13709590; doi:10.3390/sports14100468)
Supplement: Supplementary file 1 [file sports-14-00468-s001.zip › Table S3_ Baseline outcome measures completers vs. non-completers.pdf]

Supplementary Table S3. Baseline Hyperarch Fascia Training completers versus non-completers.

Outcome Measure	Completers (n = 22)	Non-completers (n = 22)	SMD
**CAIT**			
Injured Ankle	15.68 ± 4.66	14.59 ± 4.82	0.23
**FAAM**			
ADL Percent	91.16 ± 8.27	86.47 ± 12.09	0.45
ADL Global	86.73 ± 9.21	79.32 ± 11.58	0.71
Sport Percent	76.99 ± 14.09	74.86 ± 16.69	0.14
Sport Global	77.27 ± 14.43	70.68 ± 19.78	0.38
**Vertical Jump Performance**			
Jump Height (cm)	32.85 ± 13.31	33.79 ± 10.89	-0.08
Force (N)	3111.62 ± 2040.30	2433.18 ± 1325.10	0.39
Relative Force (N/kg)	41.24 ± 27.50	33.34 ± 17.92	0.34
Power (W)	4173.43 ± 3498.83	3107.17 ± 1774.23	0.38
Relative Power (W/kg)	54.68 ± 45.41	42.14 ± 22.39	0.35
Mean Velocity (m/s)	1.24 ± 0.26	1.27 ± 0.23	-0.11
Take-off Velocity (m/s)	2.49 ± 0.51	2.54 ± 0.45	-0.10
Impulse (kg*m/s)	203.94 ± 59.74	212.83 ± 97.66	-0.11
Flight Time (ms)	507.27 ± 104.87	517.18 ± 92.49	-0.10
**SF-12**			
Physical Function	95.45 ± 12.53	90.91 ± 18.17	0.29
Limits due to Physical Health	84.09 ± 35.81	81.82 ± 36.34	0.06
Limits due to Emotional Health	97.73 ± 10.66	93.18 ± 23.38	0.25
Energy/Fatigue	71.82 ± 13.32	65.45 ± 16.54	0.42
Emotional Wellbeing	78.64 ± 13.20	78.18 ± 12.96	0.04
Social Functioning	92.05 ± 11.92	85.23 ± 18.35	0.44
Pain	84.09 ± 14.53	79.55 ± 18.32	0.27
General Health	79.55 ± 14.71	78.41 ± 19.36	0.07

Data are mean ± standard deviation. SMD = standardized mean difference for completers minus non-completers; positive values indicate higher scores in completers. CAIT = Cumberland Ankle Instability Tool; FAAM = Foot and Ankle Ability Measure; ADL = Activities of Daily Living; SF-12 = Short Form Health Survey 12.
