# Supplementary material for: The Impact of Hyperarch Fascia Training on Perceived Ankle Instability and Vertical Jump Performance in Physically Active Men and Women
Source: Sports (Basel). 2026 Oct 20;14(10):468. doi: 10.3390/sports14100468 (PMC13709590; doi:10.3390/sports14100468)
Supplement: Supplementary file 1 [file sports-14-00468-s001.zip › Table S4_Hyperarch_fascia training_protocol.pdf]

Supplementary Table S4. Hyperarch Fascia Training: Protocol Details

Table S4A. Session structure

One row per exercise, in the order performed.

#	Exercise	Sets	Reps or duration	Rest between sets
1	Myofascial rolling (foot → calf → glutes)[1]	1	to fatigue / 1–2 min	continuous
2	Elevated towel curls	2	to fatigue / 1–2 min	30 second
3	Toe raises	2–3	to fatigue / 1–2 min	1min
4	Balance exercises (Two-feet → Single-foot → marbles)[2]	2–3	to fatigue / 1–2 min	1 min
5	Plyometric jumping activities and landing activities (two-feet → Single-foot)[3]	2–3	to fatigue / 1–2 min	1 min
6	Movement drills (Heels → toes → balls of feet)[4]	2–3	to fatigue / 1–2 min	1 min
7	Mental imagery[5]	2–3	2- 5 min	1 min
8	Calf raises	1-2	1-2min	1min
9	Fascia stretch	1	1-2min	

[1]Coaches used the meridians line as main targets as they integrate with body functions and starting from foot, calf, glutes, and progressing along the kinetic chain.

[2]For balance exercises, coaches started with standing on 2 feet, until the proper posture was achieved, then progressed to single leg balance with one foot while the other foot is holding a marble and moving in a different plane of motion.

[3] Plyometric jumping activities started with both feet, and progressed to single leg activity, with the instructions on form given through jumping movements.

[4] Movement drills consisted of moving 2 feet in all directions, with particular emphasis placed by coaches on moving from heel to toes. Then, once subjects were deemed by the coaches to have achieved good balance, control and rhythm in transitioning to the balls of their feet, subjects progressed to landing using only the balls of their feet.

[5] Mental imagery exercises, subjects were instructed to imagine their body as one unit and move like one, with a specific focus on moving the energy from the feet upwards to the body. Lastly,

Table S4B. Week-by-week progression

Weeks	What was performed	What changed from the previous block
Weeks 1–5	Myofascial rolling (foot → calf → glutes)[1]<br>Elevated towel curls<br>Toe raises<br>Calf raises<br>Balance exercises (Two-feet → Single-foot → marbles)[2]<br>Plyometric jumping activities and landing activities (two-feet → Single-foot)[3]<br>Mental imagery[5]<br>Calf raises	Gradually progress with more time duration per exercise and from 2 feet to single foot exercise.
Week 6-8	Myofascial rolling (foot → calf → glutes)[1]<br>Elevated towel curls<br>Toe raises<br>Calf raises<br>Balance exercises (Two-feet → Single-foot → marbles)[2]	Adding medicine ball hold for stability

Weeks	What was performed	What changed from the previous block
	Plyometric jumping activities and landing activities (two-feet → Single-foot)[3]<br>Mental imagery[5]<br>Calf raises	
Week 8-12	Myofascial rolling (foot → calf → glutes)[1]<br>Elevated towel curls<br>Toe raises<br>Calf raises<br>Balance exercises (Two-feet → Single-foot → marbles)[2]<br>Plyometric jumping activities and landing activities (two-feet → Single-foot)[3]<br>Mental imagery[5]<br>Calf raises	Adding more time for Plyometric and landing trainings

Table S4C. Progression and adaptations

Question	Answer
How was it decided a participant should progress?	Showing good adaptation of the exercise through ankle stability, balance and effortlessness.
Could a participant repeat or scale back a week?	no
If someone could not perform an exercise, what were they allowed to change?	If they cannot for example do single leg, start with double leg exercises until you build up the strength to do so.
Were any exercises substituted or dropped for individuals?	no
