# Supplementary material for: The Impact of Hyperarch Fascia Training on Perceived Ankle Instability and Vertical Jump Performance in Physically Active Men and Women
Source: Sports (Basel). 2026 Oct 20;14(10):468. doi: 10.3390/sports14100468 (PMC13709590; doi:10.3390/sports14100468)
Supplement: Supplementary file 1 [file sports-14-00468-s001.zip › Table S2_ Study Eligibility Criteria.pdf]

Supplementary Table S2: Study Eligibility Criteria.

**Inclusion Criteria**
• Gender: Male or females<br>• Age: 20–45 years old<br>• History of Chronic Ankle Sprains has led to present symptoms such as current pain and/or instability during weight-bearing activities, but no reported chronic knee, hip, or groin issues (within the past 3-months).<br>• Cumberland Ankle Instability Tool (CAIT) score ≤ 25, indicating significant instability in at least one ankle (Wright et al. 2014).<br>• Participants will be either recreationally trained, or considered trained/developmental as defined by McKay et al. (2021).<br>• Health Status: Free from chronic diseases, cardiovascular conditions, and musculoskeletal injuries unrelated to ankle sprains.<br>• Agree to maintain their usual activity levels and avoid additional treatment modalities for the duration of the study.<br>• During the program, required to cease all weightlifting (resistance exercise); as well as any other high-intensity exercise that directly aggravates the ankle.<br>• Have a iPhone smart phone XR (2018), or later model<br>• Have a desktop computer or laptop, with a camera for video conference calls and online training.<br>• Have a location with a minimum of 10ft * 10ft space for exercise movement, and appropriate ceiling height for vertical jump testing.
**Exclusion Criteria**
• History of Achilles tendon rupture or surgery within the last 12 months.<br>• Use of prescribed corticosteroids or other tendon-specific treatments within four weeks prior to the study.<br>• Current enrollment in any structured fascial or tendon rehabilitation programs.<br>• Pregnancy, breastfeeding, or plans to become pregnant during the study period.<br>• Any medical conditions that may interfere with running or exercise performance or study participation.<br>• Not willing to sign an NDA regarding the fascia training techniques used in this study<br>• Use of medical interventions, such as growth hormone or peptides, that may influence tissue adaptation to exercise.<br>• Current supplementation with common soft-tissue ingredients, such as glucosamine, chondroitin, methylsufonylmethane (MSM), or collagen protein.<br>• Presents with Ehlers-Danlos syndrome (EDS) or hypermobility History or current drug abuse

- History or current cigarette smoke (including vaping) within the past 14 days from the screening visit
- Insulin-dependent diabetes and/or metformin use
- Chronic kidney or liver disease
- Has significant concurrent illnesses (controlled or uncontrolled), such as diabetes, lupus, epilepsy, or cardiac disorders, hepatitis B/C, HIV, serious mental health illness such as dementia or schizophrenia; psychiatric hospitalization in the past two years, or other, which in the opinion of the investigator, such conditions might be aggravated as a result of treatment
- Clinically diagnosed as depressed or taking depression or anti-anxiety medication
- The investigator feels that for any reason the subject is not eligible to participate in the study
- History of uncontrolled cardiovascular disease (i.e., myocardial infarction, hypertension, hypercholesterolemia, peripheral vascular disease, other)
- Developmental disability or cognitive impairment that would preclude adequate comprehension of the informed consent form and/or ability to follow study subject requirement and/or record the necessary study measurements
- Currently has been diagnosed with cancer or has been in remission for less than 1 year
- Participation in any other investigational study within 30 days prior to consent.
